# fbst: An R package for the Full Bayesian Significance Test for testing a sharp null hypothesis against its alternative via the *e* value

**DOI:** 10.3758/s13428-021-01613-6

**Published:** 2021-09-01

**Authors:** Riko Kelter

**Affiliations:** Department of Mathematics, University of Siegen, Walter-Flex-Street 3, 57072 Siegen, Germany

**Keywords:** Full Bayesian Significance Test, *e* value, Bayesian hypothesis testing, Null hypothesis significance testing (NHST)

## Abstract

Hypothesis testing is a central statistical method in psychology and the cognitive sciences. However, the problems of null hypothesis significance testing (NHST) and *p* values have been debated widely, but few attractive alternatives exist. This article introduces the fbst R package, which implements the *Full Bayesian Significance Test (FBST)* to test a sharp null hypothesis against its alternative via the *e* value. The statistical theory of the FBST has been introduced more than two decades ago and since then the FBST has shown to be a Bayesian alternative to NHST and *p* values with both theoretical and practical highly appealing properties. The algorithm provided in the fbst package is applicable to any Bayesian model as long as the posterior distribution can be obtained at least numerically. The core function of the package provides the Bayesian evidence against the null hypothesis, the *e* value. Additionally, *p* values based on asymptotic arguments can be computed and rich visualizations for communication and interpretation of the results can be produced. Three examples of frequently used statistical procedures in the cognitive sciences are given in this paper, which demonstrate how to apply the FBST in practice using the fbst package. Based on the success of the FBST in statistical science, the fbst package should be of interest to a broad range of researchers and hopefully will encourage researchers to consider the FBST as a possible alternative when conducting hypothesis tests of a sharp null hypothesis.

## Introduction

Hypothesis testing is a widely used method in the cognitive and biomedical sciences. However, the recently experienced replication crisis troubles experimental sciences, and the underlying problems are still widely debated (Wagenmakers & Pashler, 2012; Pashler & Harris, 2012; Wasserstein et al., 2019; Haaf et al., 2019). Among the identified problems is the inappropriate use and interpretation of *p* values, which are used in combination with null hypothesis significance tests (NHST) (Benjamin & Berger, 2019; Benjamin et al., 2018; Colquhoun, 2014; 2017). As a consequence, in 2016 the American Statistical Association issued a statement about the identified problems and recommended to consider alternatives to *p* values or supplement data analysis with further measures of evidence:

“All these measures and approaches rely on further assumptions, but they may more directly address the size of an effect (and its associated uncertainty) or whether the hypothesis is correct.”

Wasserstein & Lazar (2016, p. 132)

Due to the problems with NHST and *p* values, the editors of *Basic and Applied Social Psychology* even decided to ban *p* values and NHST completely from their journal.

In the recent literature, various proposals have been made how to improve the reproducibility of research and the quality of statistical data analysis, in particular the reliability of statistical hypothesis tests. These proposals range from stricter thresholds for stating statistical significance (Benjamin et al., 2018) to more profound methodological changes (Kruschke & Liddell, 2018a; Wagenmakers et al., 2016; Morey et al., 2016). In the last category, an often-stated solution is a shift towards Bayesian data analysis (Wagenmakers et al., 2016; Kruschke & Liddell, 2018a; Kruschke et al., 2012; Ly et al., 2016a; 2016b). The advantages of such a shift include the adherence of Bayesian methods to the likelihood principle (Birnbaum, 1962), which has important implications. Some of them are the simplified interpretation and appealing properties of Bayesian interval estimates for quantifying the uncertainty in parameter estimates (Morey et al., 2016). Others are given by the independence of results of the researcher’s behavior (Kruschke & Liddell, 2018b; Berger & Wolpert, 1988; Edwards et al., 1963) as well as the ability to make (simplified) use of optional stopping (Rouder, 2014). The last property is, in particular, appealing in practical research, as it allows to stop recruiting participants and report the results based on the collected data in case they already show overwhelming evidence. Notice that this is not permitted when making use of NHST and *p* values, which can lead to financial and ethical problems, in particular in the biomedical and psychological sciences. Here, the ethical obligations, for example for patients in clinical trials, are profound.

Considering Bayesian alternatives to NHST and *p* values, the most prominent approach to Bayesian hypothesis testing is the Bayes factor, which is often attributed to Jeffreys (1935), see also Etz and Wagenmakers (2015).^1^ The Bayes factor is often advocated as a Bayesian alternative to the frequentist *p* value when it comes to hypothesis testing, in particular in the cognitive sciences and psychology (Van De Schoot et al., 2017; Wagenmakers et al., 2016; Wagenmakers et al., 2010; Ly et al., 2016b; van Doorn et al., 2019; van Dongen et al., 2019; Kelter, 2021). However, there are also other approaches like Bayesian equivalence testing based on the region of practical equivalence (ROPE) (Kruschke, 2013; 2015; Kruschke and Liddell, 2018b; Kruschke, 2018; Westlake, 1976; Kirkwood, 1981; Liao et al., 2020; Kelter, 2020a; 2020f, d) which are based on an analogy to frequentist equivalence tests (Lakens, 2017; Lakens et al., 2018). Also, there exist various other measures and alternatives to test hypotheses in the Bayesian approach, including the MAP-based *p* value (Mills, 2018), the probability of direction (PD) (Makowski et al., 2019; Makowski et al., 2019) and the Full Bayesian Significance Test (FBST) (Pereira & Stern, 1999; Stern, 2003; Madruga et al., 2001; Madruga et al., 2003; Pereira et al., 2008; Pereira & Stern, 2020; Esteves et al., 2019). In contemporary literature, there is still debate about which Bayesian measure to use in which setting for scientific hypothesis testing, and while some authors argue in favor of the Bayes factor (Wagenmakers et al., 2016; Etz & Vandekerckhove, 2016; Kelter, 2020b), there is also criticism about the focus on the Bayes factor in the cognitive sciences (Tendeiro & Kiers, 2019; Greenland, 2019). By now, comparisons of different Bayesian posterior indices are rare, but the existing results show that it is useful to consider various different Bayesian approaches to hypothesis testing depending on the research goal and study design, see Kelter (2020a), Makowski et al., (2019) and Liao et al., (2020).

In this paper, attention is directed to one specific Bayesian alternative to NHST and *p* values, the Full Bayesian Significance Test (FBST) and the *e* value, and the R package fbst is introduced. The FBST was developed over two decades ago in the statistical literature (Pereira and Stern, 1999), and since has been employed successfully in a broad range of scientific areas and applications. It is not possible to cover all of the theoretical and practical work that has been pursued concerning the FBST in the last two decades in this paper, and for a concise review, the reader is referred to Pereira and Stern (2020). The R package fbst introduced in this paper offers an intuitive and widely applicable software implementation of the FBST and the *e* value. The package has been designed to work in combination with widely used R packages for fitting Bayesian models in the cognitive sciences and psychology and offers appealing visualizations to communicate and share the results of an analysis with colleagues.

The structure of this paper is as follows: First, the underlying theory of the FBST and the *e* value is outlined. Second, details about the available functionality and software implementation of the package are provided. Subsequently, it is demonstrated with three examples of widely used statistical models in psychological research how the FBST can be applied in practice via the fbst package. Finally, a conclusion is given that draws attention to the benefits and limitations of the package and provides some ideas about future extensions. In summary, the FBST and *e* value could be an appealing Bayesian alternative to NHST and *p* values, which has been widely under-utilized by now in the cognitive and biomedical sciences. This clearly can be attributed to the dearth of accessible software implementations, one of which is presented in form of the R package introduced in this paper. The fbst package hopefully will foster critical discussion and reflection about different approaches to Bayesian hypothesis testing and allow to pursue further research to investigate the relationship between different posterior indices for significance and effect size (Kelter, 2020a; Makowski et al., 2019; Liao et al., 2020).

## The FBST and the *e* value

This section describes the statistical theory behind the FBST and the *e* value in more detail. The philosophical basis (or conceptual approach) is first described briefly, and subsequently, the necessary notation is introduced.

### Conceptual approach of the FBST

The Full Bayesian Significance Test was first introduced by Pereira and Stern (1999) more than two decades ago as a Bayesian alternative to traditional frequentist null hypothesis significance tests. It was invented to test a *sharp* (or precise) point null hypothesis *H*_0_ against its alternative *H*_1_.

Traditional frequentist approaches measure the inconsistency of the observed data with a null hypothesis *H*_0_ (Kempthorne, 1976; Cox et al., 1977). Frequentist hypothesis tests employ *p* values to order the *sample space* according to increasing inconsistency with the hypothesis. Notice, that a *p* value is defined as the probability of obtaining a result (which, of course, is located in the sample space) equal to or more extreme than the one observed under the assumption of the null hypothesis *H*_0_ (Held & Sabanés Bové, 2014). In contrast, the *e* value produced in the FBST aims at ordering the *parameter space* according to increasing inconsistency with the observed data (Pereira et al., 2008). In formulas, traditional frequentist significance tests use the *p* value to reject the null hypothesis *H*_0_:

$$ \begin{array}{@{}rcl@{}} p=Pr(x\in C|\theta_{0}) \end{array} $$

Here, *C* often is the set of sample space values $x\in \mathcal {X}$ (where $\mathcal {X}$ is the sample space) for which a test statistic $T_{\theta _{0}}$ (derived under the assumption of the null hypothesis value *𝜃*_0_) is at least as large as the test statistic value *t* calculated from the observed data. The set *C* can be interpreted as the sample space values $x\in \mathcal {X}$, which are at least as *inconsistent* with the null hypothesis *H*_0_ as the observed data. The *p* value now quantifies the evidence against *H*_0_ by calculating the probability of sample space values *x* being located precisely in this set (Casella & Berger, 2002).

The idea put forward in Pereira and Stern (1999) and Pereira et al., (2008) is simple: Instead of considering the sample space, a Bayesian should inspect the *tangential set* *T* of parameter values (which are, of course, located in the parameter space). This set consists of all parameter values which are *more consistent* with the observed data *x* than *𝜃*_0_, which is the Bayesian evidence $\overline {ev}$. Here, $\overline {ev}$ is defined as

$$ \begin{array}{@{}rcl@{}} \overline{ev} = Pr(\theta \in T|x) \end{array} $$

and $ev=1-\overline {ev}$. The quantity *ev* can be interpreted as the evidence value supporting the null hypothesis *H*_0_, while $\overline {ev}$ is interpreted as the evidence *againstH*_0_. This latter value is the probability of all parameter values *𝜃* which are *more consistent* with the data *x* than the null value *𝜃*_0_. The conceptual approach of the FBST consists, as a consequence, of constructing a duality between Bayesian theory and frequentist sampling theory. This duality is constructed between frequentist significance measures, which are based on ordering the *sample space* according to increasing inconsistency with the data, and the Bayesian *e* value, which is based on ordering the *parameter space* according to increasing inconsistency with the observed data. This conceptual basis ensures that the FBST allows a seamless transition to Bayesian data analysis for researchers who are acquainted with NHST and *p* values. The FBST produces the *e* value which can be interpreted similarly to the frequentist *p* value and little methodological changes are required. However, the consequences of the conceptual basis of the FBST are substantial: As the quantity $\overline {ev}$ is a fully Bayesian quantity, it allows statements in terms of probability to quantify the evidence. Traditional frequentist measures like *p* values do not make probabilistic statements about the parameter (because they are computed over the sample space instead of the parameter space), which is questionable as the goal of an experiment or study often is to quantify the uncertainty about a given research hypothesis, which naturally can be achieved via probability measures (Howie, 2002; Berger and Wolpert, 1988). Frequentist procedures are often interested in the “long-term” performance of a procedure, and examples are Neyman–Pearson tests where the type I error probability is controlled in expectation, but no statement about the false-positive (or false-negative) probability of the research hypothesis at hand can be made:

“Without hoping to know whether each separate hypothesis is true or false, we may search for rules to govern our behavior with regard to them, in following which we insure that, in the long run of experience, we shall not be too often wrong. Here, for example, would be such a “rule of behavior”: to decide whether a hypothesis, *H*, of a given type be rejected or not, calculate a specified character, *x*, of the observed facts; if *x* > *x*_0_ reject *H*, if *x* ≤ *x*_0_ accept *H*. Such a rule tells us nothing as to whether in a particular case *H* is true when *x* ≤ *x*_0_ or false when *x* > *x*_0_. But it may often be proved that if we behave according to such a rule, then in the long run we shall reject *H* when it is true not more, say, than once in a hundred times, and in addition we may have evidence that we shall reject *H* sufficiently often when it is false.”

(Neyman & Pearson, 1933, p. 291)

There are various situations in which such reasoning is adequate (e.g., medical tests for a disease which are repeated under approximately similar conditions a large number of times or quality control of items produced by a machine). However, experiments and studies are seldom repeated under identical or even approximately similar conditions, and one could even argue that in the biomedical and cognitive sciences this is not possible at all. In situations where probabilistic statements about a research hypothesis are desired, the Bayesian approach thus may be more appropriate, also because of the adherence to the likelihood principle (Birnbaum, 1962; Basu, 1975; Berger & Wolpert, 1988). Due to their Bayesian nature, the FBST and the *e* value also follow the likelihood principle, which brings several advantages with it:

- Researchers can use optional stopping. This implies that they are allowed to stop recruiting participants or even abort an experiment and readily report the results when only a fraction of the data already shows overwhelming evidence for or against the hypothesis under consideration (Edwards et al., 1963; Rouder, 2014). Of course, frequentist statisticians can also use optional stopping, if the test statistic is changed accordingly when using a different stopping rule. However, this complicates the analysis and introduces a “researcher degree of freedom”, as the stopping rule used can change the outcome of a hypothesis test. Partially, this also applies to the Bayesian approach, but as long as the stopping rule is noninformative (that is, the stopping rule provides no information about the parameter), the *stopping rule principle* – see Berger & Wolpert (1988, Chapter 4) – guarantees that the stopping rule does not influence the obtained results (Hendriksen et al., 2020).
- Censored data (which are often observed in longitudinal studies or clinical trials in the cognitive sciences and psychology) can be interpreted easily (Berger & Wolpert, 1988). The likelihood contribution of a single observation in a study where no censoring was possible is equal to the likelihood contribution of a single observation in a study where censoring is possible but did not occur (for the single observation considered). This simplifies the analysis and interpretation of statistical models which include censoring mechanisms, see Berger & Wolpert (1988, Chapter 4).
- As highlighted by Edwards et al., (1963), Wagenmakers et al., (2016), and Kruschke (2018), the result of a hypothesis test (in this case, the FBST), is not influenced by the researchers’ behavior. This last property is substantial for improving the reliability of research in the cognitive sciences and psychology, see McElreath and Smaldino (2015).

### Statistical theory of the FBST

In this section, the necessary mathematical notation for a rigid understanding of the FBST is introduced. The FBST can be used with any standard parametric statistical model, where $\theta \in {{\varTheta }} \subseteq \mathbb {R}^{p}$ is a (vector valued) parameter of interest, *p*(*x*|*𝜃*) is the model likelihood and *p*(*𝜃*) is the prior density for the parameter *𝜃* of interest. A sharp (or expressed equivalently, precise) hypothesis *H*_0_ makes a statement about the parameter *𝜃*: Specifically, the null hypothesis *H*_0_ states that *𝜃* lies in the so-called *null set* ${{\varTheta }}_{H_{0}}$. For simple point null hypotheses like *H*_0_ : *𝜃* = *𝜃*_0_, which are often used in practice, this null set consists of the single parameter value *𝜃*_0_ so that the null set can be written as ${{\varTheta }}_{H_{0}} = \{\theta _{0} \}$. As detailed in the previous section, the conceptual approach of the FBST is to state the Bayesian evidence against *H*_0_, the *e* value. This value is the proposed Bayesian replacement of the traditional *p* value. To construct the *e* value, Pereira et al., (2008) introduced the posterior *surprise functions*(*𝜃*) which is defined as follows:

1 $$ \begin{array}{@{}rcl@{}} s(\theta):=\frac{p(\theta|x)}{r(\theta)} \end{array} $$

The surprise function *s*(*𝜃*) is the ratio of the posterior distribution *p*(*𝜃*|*x*) and a suitable *reference function* *r*(*𝜃*). The first thing to note is that two important special cases are given by a flat reference function *r*(*𝜃*) = 1 or any prior distribution *p*(*𝜃*) for the parameter *𝜃*. First, when a flat reference function is selected the surprise function recovers the posterior distribution *p*(*𝜃*|*x*). Second, when any prior distribution is used as the reference function, one can interpret parameter values *𝜃* with a surprise function value *s*(*𝜃*) ≥ 1 as being corroborated by the observed data *x*. In contrast, parameter values *𝜃* with a surprise function *s*(*𝜃*) < 1 indicate that they have not been corroborated by the data. The next step is to calculate the supremum *s*^∗^ of the surprise function *s*(*𝜃*) over the null set ${{\varTheta }}_{H_{0}}$.

$$ \begin{array}{@{}rcl@{}} s^{*}:=s(\theta^{*})=\sup\limits_{\theta \in {{\varTheta}}_{H_{0}}}s(\theta) \end{array} $$

This supremum is subsequently used in combination with the tangential set, which has been introduced in the last section. Pereira et al., (2008) defined

2 $$ \begin{array}{@{}rcl@{}} T(\nu):=\{\theta \in {{\varTheta}}|s(\theta)\leq \nu \} \end{array} $$

and the tangential set $\overline {T}(\nu )$ to the sharp null hypothesis *H*_0_ is then given as follows:

3 $$ \begin{array}{@{}rcl@{}} \overline{T}(\nu):={{\varTheta}} \setminus T(\nu) \end{array} $$

When setting *ν* = *s*^∗^, the tangential set $\overline {T}(\nu )$ has its unique interpretation which has been discussed in the previous section: While *T*(*s*^∗^) includes all parameter values *𝜃* which attain smaller or equal surprise as the supremum value $s^{*} \overline {T}(s^{*})$ includes all parameter values *𝜃* which attain a *larger* surprise value than the supremum *s*^∗^ of the null set.

The final step to obtain the *e* value, the Bayesian evidence against *H*_0_, is to make use of the *cumulative surprise function* *W*(*ν*)

4 $$ \begin{array}{@{}rcl@{}} W(\nu):={\int}_{T(\nu)}p(\theta|x)d\theta \end{array} $$

The cumulative surprise function *W*(*ν*) is simply an integral of the posterior density *p*(*𝜃*|*x*) over all parameter values with surprise function values *s*(*𝜃*) ≤ *ν*. Setting *ν* = *s*^∗^, the cumulative surprise function *W*(*s*^∗^) becomes the integral of the posterior *p*(*𝜃*|*x*) over *T*(*s*^∗^). This is the integral of the posterior *p*(*𝜃*|*x*) over all parameter values which have a surprise function value *s*(*𝜃*) ≤ *s*^∗^. The *e* value is then given as

5 $$ \begin{array}{@{}rcl@{}} \overline{\text{ev}}(H_{0}):=\overline{W}(s^{*}) \end{array} $$

**Fig. 1:**
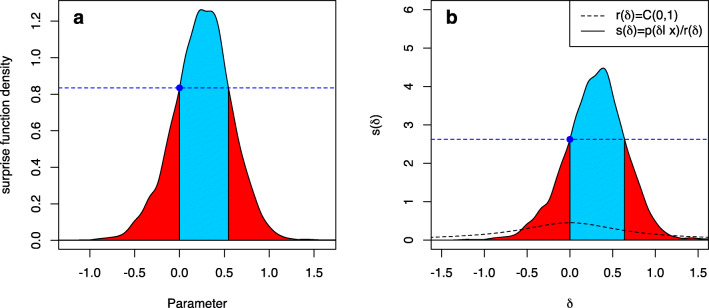
The FBST and the *e* value $\overline {\text {ev}}(H_{0})$ against *H*_0_ : *δ* = 0 in a Bayesian two-sample *t* test, where *δ* is the effect size. **a** A flat reference function *r*(*δ*) = 1 is used, and the *solid line* is the resulting posterior distribution *p*(*δ*|*x*) after observing the data. The supremum over the null set *s*^∗^ = 0 is visualized as the blue point. The *blue shaded area* corresponds to the cumulative surprise function $\overline {W}(0)$, which is the integral over the tangential set $\overline {T}(0)$ of *H*_0_ : *δ* = 0. This is the *e* value $\overline {\text {ev}}(H_{0})$ against *H*_0_. The *red area* is the integral *W*(0) over *T*(0), and equals the *e* value ev(*H*_0_) in favor of *H*_0_ : *δ* = 0. **b** The same situation as in (**a**), but now a Cauchy *C*(0,1) prior has been used as reference function *r*(*δ*)

Here $\overline {W}(\nu ):=1-W(\nu )$. Figure 1a visualizes the FBST and the *e* value $\overline {\text {ev}}(H_{0})$. The solid line shows the posterior distribution *p*(*δ*|*x*) of the effect size *δ* after observing the data *x*, and is produced by a Bayesian two-sample *t* test (Kelter, 2020d). A flat reference function *r*(*δ*) = 1 was selected in Fig. 1a. The supremum over the null set ${{\varTheta }}_{H_{0}}=\{0\}$ is *s*^∗^ = *s*(0), shown as the blue point. The horizontal blue dashed line visualizes the boundary between *T*(0) and $\overline {T}(0)$, and values with posterior density *p*(*δ*) > *p*(0) are located in $\overline {T}(0)$, while values with *p*(*δ*) ≤ *p*(0) are located in *T*(0). The blue shaded area is the cumulative surprise function $\overline {W}(0)$, which is the integral over the tangential set $\overline {T}(0)$ against *H*_0_ : *δ* = 0. This is the *e* value $\overline {\text {ev}}(H_{0})$ against *H*_0_, the Bayesian evidence against the sharp null hypothesis. The red shaded area is the integral *W*(0) over *T*(0), which equals the *e* value ev(*H*_0_) in favor of *H*_0_ : *δ* = 0. Figure 1b shows the same situation, but now the reference function is selected as a wide Cauchy prior *C*(0,1), so that the surprise function becomes

$$ \begin{array}{@{}rcl@{}} s(\delta)=p(\delta|x)/c(\delta) \end{array} $$

where *c*(*δ*) is the p.d.f. of the *C*(0,1) Cauchy distribution. Although the situation seems similar to Fig. 1a, the scaling on the *y*-axis now is different. Also, the evidence has changed based on the new surprise function and the interpretation of the surprise function has changed, too. While in Fig. 1a, the surprise function could be interpreted as the posterior distribution, now it is interpreted as follows: If one assumes a Cauchy prior *C*(0,1) on the effect size *δ*, then parameters with a surprise function value *s*(*δ*) ≥ 1 can be interpreted as being corroborated by the data. Parameter values with a surprise function *s*(*δ*) < 1 are interpreted as not being corroborated by the data.

Pereira and Stern (1999) formally defined the *e* value ev(*H*_0_) in *support* of *H*_0_ as

6 $$ \begin{array}{@{}rcl@{}} \text{ev}(H_{0}):=1-\overline{\text{ev}}(H_{0}) \end{array} $$

but notice that one cannot interpret this value as evidence *againstH*_1_. This can be attributed to the fact that *H*_1_ is not even a sharp hypothesis, see Definition 2.2 in Pereira et al., (2008).

It is crucial to note that by definition of the FBST it is not possible to utilize the *e* value ev(*H*_0_) to *confirm* the null hypothesis *H*_0_. The FBST formally is defined as the procedure which rejects *H*_0_ when ev(*H*_0_) is small, or equivalently, when $\overline {\text {ev}}(H_{0})$ is large (Pereira et al., 2008, Definition 2.3, Definition 4.2). Therefore, it is by definition not possible to accept *H*_0_ based on ev(*H*_0_) or $\overline {\text {ev}}(H_{0})$. Even if one would be tempted to extend the definition of the FBST to allow for such acceptance of the null hypothesis whenever ev(*H*_0_) is large enough (or equivalently, when $\overline {\text {ev}}(H_{0})$ is small enough), problems with interpretation can arise: Kelter (2020a) showed that even when data are distributed as specified under *H*_0_ the *e* value ev(*H*_0_) does not necessarily converge to one for $n\rightarrow \infty $. Details are provided in Kelter (2021a) and Ly and Wagenmakers (2021). This troubles the identification of a threshold based on which *H*_0_ is accepted. However, the FBST can be generalized into an extended framework, which then allows for hypothesis confirmation and itself is an active topic of ongoing research (Esteves et al., 2019). In summary, the *e* value ev(*H*_0_) can only be used to reject *H*_0_ if ev(*H*_0_) is sufficiently small, and then also asymptotic arguments are available (Pereira et al., 2008, Section 5). Borges and Stern (2007) showed that the *e* value converges in distribution to a Chi-square cumulative distribution function

7 $$ \begin{array}{@{}rcl@{}} \overline{\text{ev}}(H_{0}) \xrightarrow[n \rightarrow \infty ]{d} F_{k}(||m-M||^{2}) \end{array} $$

where *k* is the dimension of the parameter space *Θ*, *M* is the posterior mode calculated over the entire parameter space *Θ* and *m* is the posterior maximum over ${{\varTheta }}_{H_{0}}$. *F*_*k*_(⋅) denotes the cumulative distribution function of the ${\chi _{k}^{2}}$-distribution with *k* degrees of freedom. Now, as $\text {ev}(H_{0})=1-\overline {\text {ev}}(H_{0})$, one can approximate ev(*H*_0_) as

8 $$ \begin{array}{@{}rcl@{}} \text{ev}(H_{0})\approx 1-F_{k}(||m-M||^{2}) \end{array} $$

in large samples, that is, as the upper tail of the cumulative ${\chi _{k}^{2}}$ distribution function starting from the point ||*m* − *M*||^2^.

There are two options for using asymptotic arguments now: A frequentist one and a Bayesian one. The frequentist *p* value associated with the Bayesian evidence in support of *H*_0_ is based on the asymptotic distribution of the likelihood ratio statistic and an analogy between the tangential set and the likelihood ratio statistic (for details, see Pereira et al., (2008)). It is calculated as the superior tail of the $\chi _{k-h}^{2}$ density with *k* − *h* degrees of freedom, starting from − 2*λ*(*m*_0_). Here, *k* is the dimension of the parameter space *Θ* and *h* is the dimension of the null set ${{\varTheta }}_{H_{0}}$. The quantity *m*_0_ is the observed value and $\lambda (t)=\ln l(t)$ is the logarithm of the relative likelihood function, where *l*(*t*) = *L*(*t*)/*L*(*M*) is the relative likelihood. Denoting *F*_*k*−*h*_ as the Chi-square distribution’s cumulative distribution function with *k* − *h* degrees of freedom, the frequentist *p* value associated with the Bayesian *e* value ev(*H*_0_) is then computed as

9 $$ \begin{array}{@{}rcl@{}} pv_{0}=1-F_{k-h}(-2\lambda(m_{0})) \end{array} $$

This latter *p* value has a frequentist interpretation. The second option is a Bayesian *p* value based on Equation (7), which can be expressed as

10 $$ \begin{array}{@{}rcl@{}} ev_{0} \approx 1- F_{k}(||m_{0}-M_{0}||^{2}) \end{array} $$

*ev*_0_ can be interpreted as a Bayesian significance value, which can be used after calculating the difference of 1 and *F*_*k*_(||*m*_0_ − *M*_0_||^2^), which is obtained from the quantiles of the *F*_*k*_ distribution. As a cumulative distribution function, *F*_*k*_ ∈ [0,1], so 1 − *F*_*k*_(||*m*_0_ − *M*_0_||^2^) ∈ [0,1] too. If *ev*_0_ ≈ 1 − *F*_*k*_(||*m*_0_ − *M*_0_||^2^) < 0.05, this implies that the probability of obtaining an e value as small as ev(*H*_0_) or even smaller is less than 0.05, and one could reject *H*_0_. One can rephrase this also as follows: 1 − *F*_*k*_(||*m*_0_ − *M*_0_||^2^) < 0.05 is equivalent to *F*_*k*_(||*m*_0_ − *M*_0_||^2^) > 0.95. As $F_{k}(||m_{0}-M_{0}||^{2}) \approx \overline {ev}_{0} =1-ev_{0}$ (compare Equation (10)), this means that the probability inside the tangential set – or against *H*_0_ – which is given by $\overline {ev}_{0}$ is > 0.95. Thus, *H*_0_ should be rejected.

Consequently, after observing *m*_0_ and *M*_0_ one only needs to calculate the Euclidian distance *d*_0_ = ||*m*_0_ − *M*_0_||^2^ and the difference between 1 and the value of the ${\chi _{k}^{2}}$ distribution’s cumulative distribution function *F*_*k*_ of this distance is a large sample approximation for the Bayesian *p* value *ev*_0_. Based on a threshold (like 0.05), one can decide to reject the null hypothesis *H*_0_ : *𝜃* = *𝜃*_0_ or not. Notice that the difference between *pv*_0_ and *ev*_0_ is merely that the Bayesian *p* value *ev*_0_ is based on the asymptotic normality of the posterior distribution (Held and Sabanés Bové, 2014; van der Vaart, 1998), while the frequentist *p* value *pv*_0_ is based on the asymptotic distribution of − 2*λ*(*m*), which according to Wilk’s theorem is the $\chi _{k-h}^{2}$ distribution with *k* − *h* degrees of freedom (Pereira et al., 2008, p. 90).

However, if a *p* value is required that is closest to the frequentist *p* value in interpretation, one should use the standardized *e* value sev(*H*_0_), as defined in Borges & Stern (2007, Section 2.2) and in Pereira & Stern (2020, Section 3.3). The standardized *e* value is defined as:

$$ \begin{array}{@{}rcl@{}} \overline{\text{sev}}(H_{0})=F_{k-h}(F^{-1}_{k}(\overline{\text{ev}})) \end{array} $$

Here, $F^{-1}_{k}$ is the quantile function of the cumulative distribution function of the ${\chi _{k}^{2}}$ distribution with *k* degrees of freedom. $\overline {\text {sev}}(H_{0})$ can, as a consequence, be interpreted as the probability of obtaining less evidence than $\overline {\text {ev}}(H_{0})$ against the null hypothesis *H*_0_. Defining

$$ \begin{array}{@{}rcl@{}} \text{sev}(H_{0})=1-\overline{\text{sev}}(H_{0}) \end{array} $$

sev(*H*_0_) can then be interpreted as the probability of obtaining $\overline {\text {ev}}(H_{0})$ or more evidence against *H*_0_, which is closely related to the interpretation of a frequentist *p* value. If sev(*H*_0_) is small, this implies that the probability of obtaining even more evidence against the null hypothesis *H*_0_ than the evidence against it observed, namely $\overline {\text {ev}}(H_{0})$, is small. As a consequence, one can reject *H*_0_. However, the *p* value operates in the sample space while the standardized *e* value operates in the parameter space. The standardized *e* value can be used as a Bayesian replacement of the frequentist *p* value, while being very similar in interpretation. For theoretical properties of sev(*H*_0_), see Borges and Stern (2007) and Pereira and Stern (2020).

In the examples below, the Bayesian evidence against *H*_0_, the *e* value $\overline {\text {ev}}(H_{0})$ is reported and also the standardized *e* values sev(*H*_0_) are given. The *e* value $\overline {\text {ev}}(H_{0})$ is fully Bayesian and makes no use of any asymptotic arguments, while the standardized *e* value sev(*H*_0_) uses the asymptotic normality of the posterior, the well-known Bernstein-von-Mises theorem (van der Vaart, 1998). Note that in small samples, the standardized *e* value sev(*H*_0_) may thus be unreliable.

To close this section, some information is provided how to select or justify the reference function in practice. The reference function is arguably a critical aspect on which the justification of the whole procedure hinges. However, from a theoretical perspective there are two rules of thumb which are helpful:

1. The reference function should be equal (or at least similar) to the model prior. The reason is that the tangential set should express the relative surprise *p*(*𝜃*|*x*)/*r*(*𝜃*), which naturally makes sense for the a priori beliefs *r*(*𝜃*) = *p*(*𝜃*), where *p*(*𝜃*) is the prior distribution on the parameter *𝜃*. If the reference function is selected differently, caution is necessary when interpreting the results: In the context of drug development, one could choose the reference function $r(\theta )=p^{\prime }(\theta |x)$ where $p^{\prime }(\theta |x)$ is the posterior density for the parameter *𝜃* (e.g., the effect size) of an existing drug. This means that although one uses prior distribution *p*(*𝜃*) to obtain the posterior *p*(*𝜃*|*x*) when studying the new drug, one compares the new posterior to the old posterior of the existing drug. When the new drug is better, the surprise function $s(\theta )=p(\theta |x)/p^{\prime }(\theta |x)$ should be larger for *𝜃*≠ 0 (and smaller for *𝜃* = 0). If the sharp null hypothesis *H*_0_ : *𝜃* = 0 is chosen, the tangential set $\bar {T}(s^{*})=\{\theta \in {{\varTheta }}: s(\theta )>s^{*}\}=\{\theta \in {{\varTheta }}: p(\theta |x)/p^{\prime }(\theta |x) > p(0|x)/p^{\prime }(0|x)\}$ becomes the set for which the relative surprise $p(\theta |x)/p^{\prime }(\theta |x)$ is larger than the relative surprise one would expect to observe if there were no effect. That is, the ratio between the posterior *p*(*𝜃*|*x*) of the new drug and $p^{\prime }(\theta |x)$ of the old drug should be at least as large as the ratio $p(0|x)/p^{\prime }(0|x)$ we would observe when both drugs would be ineffective. If this tangential set is large, this implies that a lot of probability mass indicates that the improvement of the new drug – expressed as the increase in the ratio between both densities $p(\theta |x)/p^{\prime }(\theta |x)$ – is larger than the “white noise” we would expect to observe under no effects of both drugs. This example illustrates that using different reference functions offers high flexibility, but simultaneously complicates interpretation.
2. As a second rule of thumb, it is recommended to use weakly informative priors (McElreath, 2020) and conduct a sensitivity analysis similar to the ones used for Bayes factors (Kelter, 2020b) to study the influence of a reference function and prior. This helps to avoid unstable results which strongly depend on the reference function (or model prior).

## Overview and functionality of the fbst package

The centerpiece of the fbst package is the fbst() function, which is used to perform the FBST. In addition to the fbst() function, the package provides customized summary() and plot() functions which allow users to print the results of a FBST or obtain a visualization of their results to communicate and share the results. The fbst() function has the following structure: 
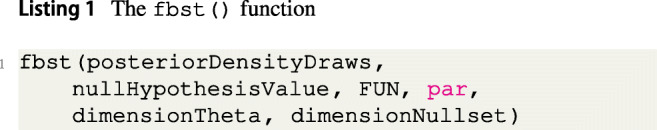
 Here, posteriorDensityDraws needs to be a numeric vector holding the posterior parameter draws obtained via MCMC or any other numerical method of choice.^2^ The argument nullHypothesisValue is the value specified in the null hypothesis *H*_0_ : *𝜃* = *𝜃*_0_, and dimensionTheta is the dimension of the parameter space *Θ*. dimensionNullset is the dimension of the null set ${{\varTheta }}_{H_{0}}$, and FUN and par are additional arguments which only need to be specified when a user-defined reference function *r*(*𝜃*) is desired. In general, FUN should be the name of the reference function which should be used and par should be a list of parameters which this reference function utilizes (e.g., the location and scale parameters when the reference function is a Cauchy prior). Details will be given in the examples below.

The fbst() function returns an object of the class fbst, which stores several useful details and the results of the conducted FBST. To obtain a concise summary of the FBST, the summary() function of the class fbst can be used. To visualize the FBST, the plot() function of the fbst class can be used. Details are provided in the examples below.

From an algorithmic perspective, the fbst package proceeds via the following steps when computing the e value via the fbst() function:

1. Based on the posterior parameter samples posteriorDensityDraws, the posterior density *p*(*𝜃*|*x*) is estimated via a Gaussian kernel density estimator, resulting in a posterior density estimate $\hat {p}(\theta |x)$. The Gaussian kernel is used due to well-known Bayesian asymptotics of posterior distributions, the Bernstein-von-Mises theorem (Held & Sabanés Bové, 2014).
2. Based on this posterior density estimate $\hat {p}(\theta |x)$, the surprise function *s*(*𝜃*) is estimated (i) as the posterior density estimate $\hat {p}(\theta |x)$ if no arguments FUN and par are supplied so that a flat reference function *r*(*𝜃*) = 1 is used as default, or (ii) as the ratio $\hat {p}(\theta |x)/r(\theta )$ if arguments FUN and par are supplied. The result is a surprise function estimate $\hat {s}(\theta )$.
3. The surprise function estimate $\hat {s}(\theta )$ is evaluated at the null hypothesis value supplied via the argument nullHypothesisValue, resulting in the value $\hat {s}_{0}$.
4. The *e* value $\overline {\text {ev}}(H_{0})$ is computed via numerical integration of the posterior density estimate $\hat {p}(\theta |x)$ over the tangential set $\overline {T}(H_{0})$, which is determined via a linear search algorithm on the vector posteriorDensityDraws by including all values *𝜃* which fulfill the condition $\hat {s}(\theta )>\hat {s}_{0}$.
5. The *p* value associated with the *e* value ev(*H*_0_) in favor of the null hypothesis *H*_0_ and the standardized *e* values sev(*H*_0_) are computed.

In summary, the FBST is based only on simple numerical optimization and integration which makes it a computationally cheap option. This is a benefit, in particular, when the parameter space *Θ* is high-dimensional (Pereira & Stern, 2020; Stern, 2003; Kelter, 2020a; 2021). Also, the presence of nuisance parameters does not trouble the computation unlike in the case of the Bayes factor, as computing the marginal likelihoods can quickly become difficult then (Stern, 2003).

## Example 1: Two-sample Bayesian *t* test

As a preliminary note, all analyses can be reproduced by following the provided code.^3^. To demonstrate how to use the fbst package, the two-sample *t* test is used, which is a widely used statistical model in the cognitive sciences (Nuijten et al., 2016; Kelter, 2021b). The two-sample Bayesian *t* test of Rouder et al., (2009) is employed with simulated data. To use the FBST, one first needs a sample of posterior draws which in this case is obtained via the BayesFactor package of Morey and Rouder (2018). Note that in general, there are multiple options available to obtain the required posterior draws: Examples are the Hamiltonian Monte Carlo sampler Stan^4^ and the rstanarm package (Goodrich et al., 2020). Another popular option is the brms package of Bürkner (2017, 2018). The recommended medium Cauchy prior $C(0,\sqrt {2}/2)$ was assigned to the effect size *δ*. Observations in the first group were simulated as $\mathcal {N}(0,1.7)$, and observations belonging to the second group were generated from the $\mathcal {N}(0.8,1.7)$ distribution. As a consequence, the resulting true effect size *δ* according to Cohen (1988) is given as

$$ \begin{array}{@{}rcl@{}} \delta = \frac{0-0.8}{\sqrt{(1.7^{2}+1.7^{2})/2}} = -0.47 \end{array} $$

which equals a small effect size. The code to simulate the data is given in Listing 2.

**Figure:**
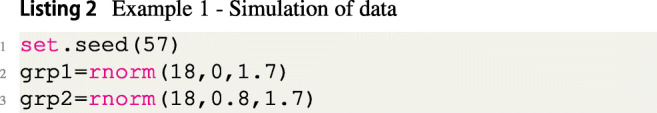

**Fig. 2:**
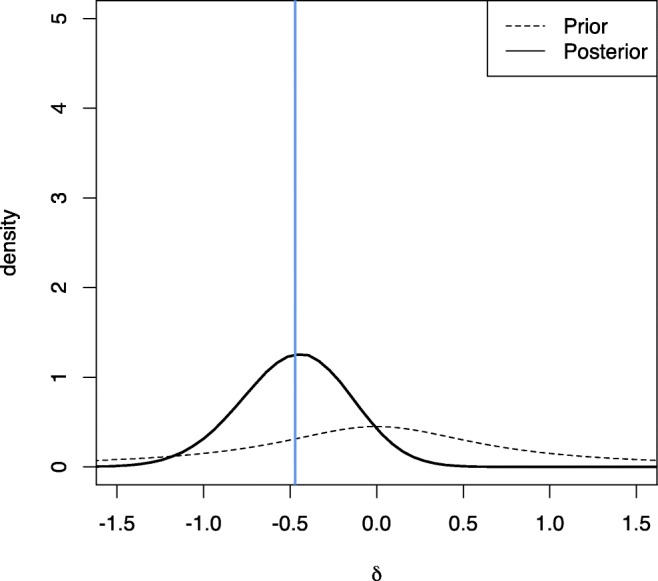
Prior-posterior plot for Example 1

The corresponding Bayes factor *BF*_10_ for the alternative hypothesis *H*_1_ : *δ*≠ 0 against the null hypothesis *H*_0_ : *δ* = 0 is given as *BF*_10_ = 1.06, which does not indicate evidence worth mentioning according to Jeffreys (1961) or van Doorn et al., (2019). The slight favor towards *H*_1_ can be attributed to the medium Cauchy prior used, which centers the prior probability mass closely around small effect sizes (and no effect, too). As a consequence, although we know that there is a small effect, the Bayes factor is slightly shrunken by the prior towards the value 1. Figure 2 shows a prior-posterior plot for the example. The code to compute the Bayes factor is given in Listing 3. Note that in Listing 3 the posterior MCMC draws produced by the ttestBF function in the BayesFactor package are stored in the variable p. Here, we could equally well use a different package like the brms package of Bürkner (2017) or even use a different sampler like Stan via the rstanarm package (Goodrich et al., 2020) to obtain these samples.

**Figure:**
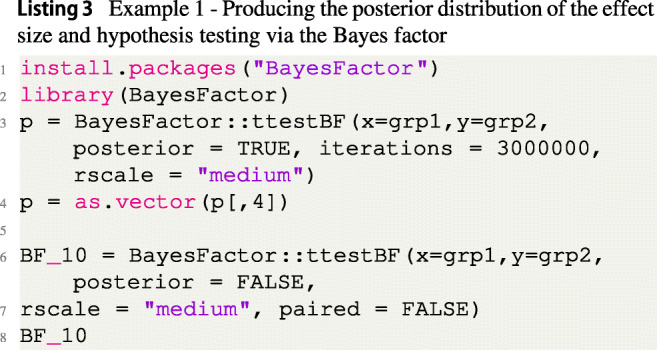

To perform the FBST and compute the *e* value, we first install and load the R package from CRAN by executing the code in Listing 4.

**Figure:**
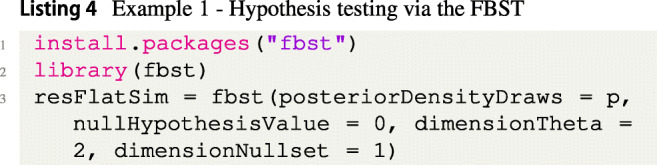

Note that in the example, the parameter space *Θ* consists of two parameters: The effect size *δ*≠ 0 and the standard deviation *σ*^2^ > 0. The null set ${{\varTheta }}_{H_{0}}$ is one-dimensional, as in *H*_0_, *δ* = 0 and *σ*^2^ > 0. As a consequence, the argument dimensionTheta is therefore set to dimensionTheta= 2. The null set ${{\varTheta }}_{H_{0}}$ is one-dimensional so that dimensionNullset = 1. The object stored in the variable resFlatSim is an object of the class fbst, which stores several values used in the summary() and plot() functions of the package. These are available to communicate and visualize the results of the FBST. For example, one can access the *e* value $\overline {\text {ev}}(H_{0})$ as follows (see Listing 5):

**Figure:**
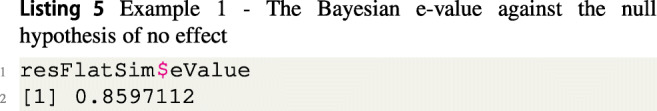

Instead of accessing each attribute manually, to obtain a summary of the FBST and print the relevant quantities the summary() function of the fbst package provides a more convenient option: 
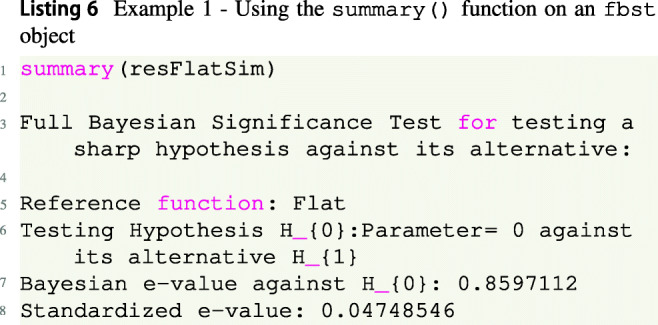
 Based on the results, we can see that there is some evidence against the null hypothesis according to the Bayesian *e* value $\overline {\text {ev}}(H_0)$ against *H*_0_ (compare Equation (5)). The standardized *e* value sev(*H*_0_) ≈ 0.047 < 0.05 is significant based on a threshold of 0.05 and indicates that the null hypothesis *H*_0_ can be rejected. Note that when a *p* value is used for hypothesis testing, it is recommended to use the standardized *e* value (Borges and Stern, 2007; Pereira & Stern, 2020) instead of *ev*_0_ or *pv*_0_, so one would reject the null hypothesis *H*_0_ : *δ* = 0 in this case. However, it is also possible to use only the Bayesian evidence $\overline {\text {ev}}(H_0)$ against *H*_0_ without any *p* value to quantify the evidence continuously. As only 18 observations are observed in each group, this may be the preferred choice here. One can conclude that 85.97% of the posterior’s probability mass indicate evidence against the null hypothesis.

The above shows that reporting and interpreting the e value is relatively straightforward: If a Bayesian interpretation is preferred, the Bayesian *e* value $\overline {\text {ev}}(H_0)$ should be reported directly, in this case, $\overline {\text {ev}}(H_0)=0.8597$. This can be interpreted as follows: 85.97% of the posterior probability mass (notice that the reference function was flat) have a larger posterior density value than the posterior density value at the null hypothesis value *δ* = 0. As a consequence, the majority of the posterior probability mass indicates evidence for values *𝜃*≠ 0 and therefore evidence against *H*_0_ : *δ* = 0.

If a significance value similar to the frequentist *p* value is desired, the standardized *e* value should be reported, which is given as sev(*H*_0_)= 0.047 in the example. This can be interpreted as the probability of obtaining 85.9*%* or more evidence against the null hypothesis *H*_0_ : *δ* = 0. As this probability is quite small, the standardized *e* value can be used to reject the null hypothesis, e.g., based on a predetermined threshold like sev(*H*_0_)= 0.047 < 0.05.

For more details on differences in the inferential foundations and interpretation between the *e* value, *p* value, and the Bayes factor (as well as multiple other Bayesian posterior indices), the interested reader is referred to Kelter (2020a).

**Fig. 3:**
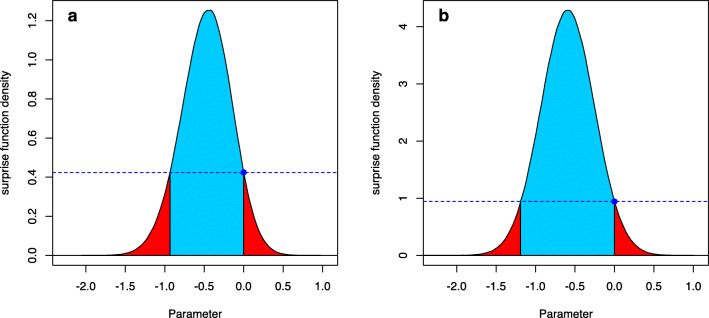
**a** Visualization of the FBST for the Bayesian two-sample *t* test in Example 1 using a flat reference function *r*(*δ*) = 1; **b** Visualization of the FBST for the Bayesian two-sample *t* test in Example 1 using a medium Cauchy prior as reference function $r(\delta )=C(0,\sqrt {2}/2)$

To visualize the results, the plot() function of the fbst package is used: 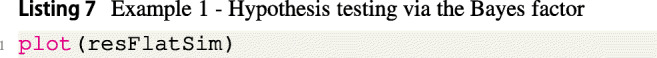 The result is shown in Fig. 3a: The blue shaded area under the surprise function (which is by default the posterior distribution, that is, a flat reference function *r*(*δ*) = 1 is used by default by the fbst() function) is the Bayesian evidence against *H*_0_, the *e* value $\overline {\text {ev}}(H_0)\approx 0.8597$ (compare Listing 6). The red shaded area is the *e* value ev(*H*_0_) in favor of *H*_0_, which is *ev*(*H*_0_) ≈ 1 − 0.8597 = 0.1403.

Instead of a flat reference function *r*(*δ*) = 1, one could also use a more reasonable prior distribution. For example, as small to medium effect sizes are to be expected in the cognitive sciences and psychology, Rouder et al., (2009) recommended to use a medium Cauchy prior $C(0,\sqrt {2}/2)$ as a default prior on the effect size. To see if parameter values *δ* have been corroborated (compared to this prior assumption) by observing the data, on can use this prior as the reference function $r(\delta )=C(0,\sqrt {2}/2)$, and the resulting surprise function is shown in Fig. 3b. The code to produce the FBST based on a Cauchy reference density is given in Listing 8:

**Figure:**
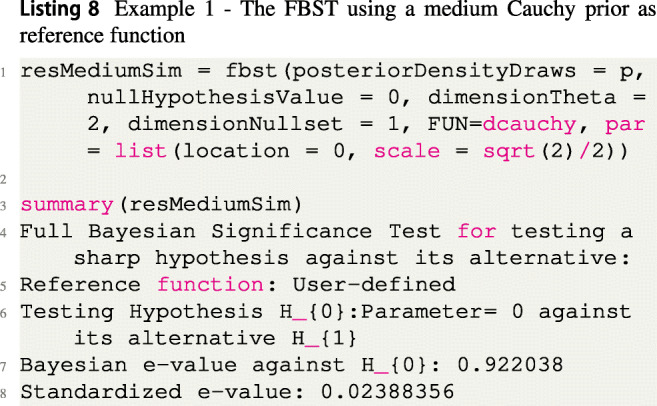

There, the FUN argument is supplied with the name of the density to be used and the par argument is supplied with a list of arguments for this density. As the Cauchy distribution has a location and scale parameter, these are supplied here. Notice that the blue point which indicates the surprise function value *s*(0) of the null hypothesis parameter *δ* = 0 is not larger than one. This means that the null hypothesis value has not been corroborated by the data. However, most parameter values in the tangential set have been corroborated by the data, and all of them have been corroborated more by the data than the null value *δ* = 0.

Based on the continuous quantification, there is again strong evidence against the null hypothesis when changing the reference function to a medium Cauchy prior: More than 90% of the posterior distribution’s parameter values attain a larger surprise function value than the null hypothesis value. The resulting standardized *e* value sev(*H*_0_) is also significant based on a threshold of 0.05.

## Example 2: Directional two-sample Bayesian *t* test

Example 1 showed how to apply the FBST in the setting of the Bayesian two-sample *t* test. Example 2 is a slight modification of Example 1. Instead of testing a two-sided hypothesis, now a directional hypothesis is considered and it is shown how such a hypothesis can easily be tested via the fbst package, too. Therefore, data of Moore et al., (2012) is used which provides the reading performance of two groups of pupils: One control group and a treatment group which was given directed reading activities. The data are freely available in the built-in data library of the open-source software JASP^5^. Interest lies in testing the hypothesis *H*_0_ : *δ* < 0, which is equivalent to the hypothesis *H*_0_ : *μ*_1_ < *μ*_2_, where the measured quantity is the performance of pupils in the degree of reading power test (DRP) (Moore et al., 2012).

First, data are saved in a .csv-file (which is called DirectedReadingActivities.csv in Listing 9), the working directory is set and then data is loaded^6^:

**Figure:**
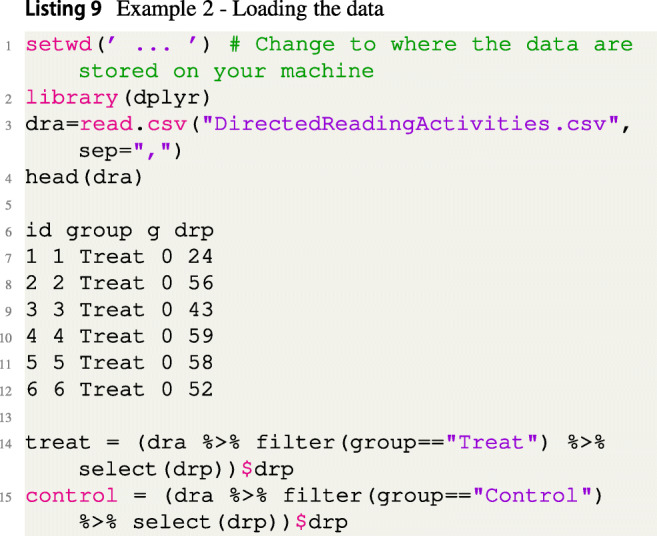

The code to perform a standard hypothesis test based on the Bayes factor is given in Listing 10, which results in *BF*_10_ = 4.32, indicating moderate evidence for the alternative *H*_1_ : *δ* < 0 according to Jeffreys (1961).

**Figure:**
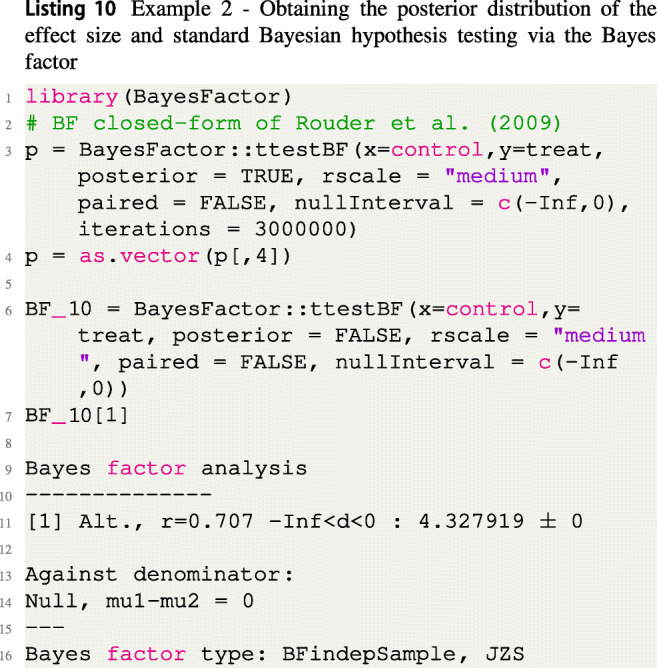

The code to perform the FBST with a flat reference function *r*(*δ*) = 1 is given in Listing 11:

**Figure:**
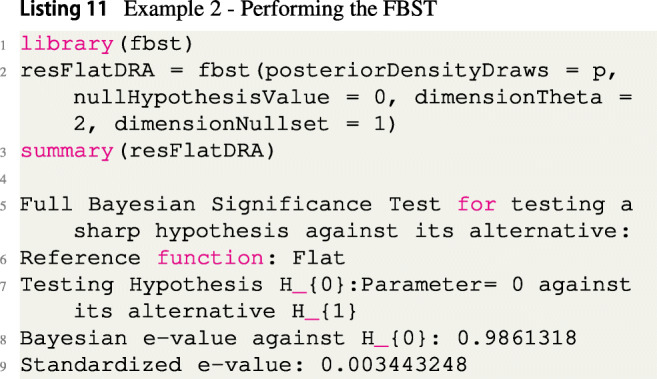

**Fig. 4:**
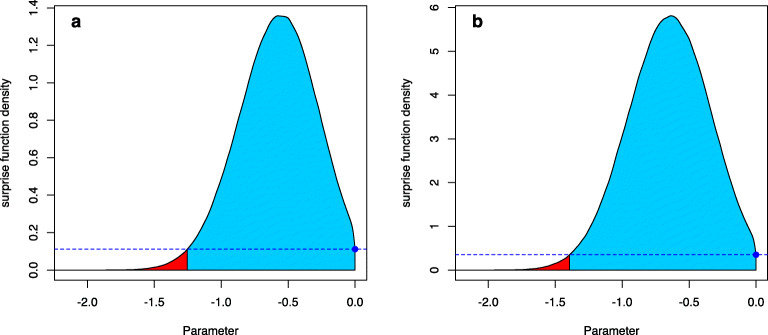
**a** visualization of the FBST in Example 2 for the Bayesian two-sample *t* test for testing *H*_0_ : *δ* = 0 against the one-sided hypothesis *H*_1_ : *δ* < 0, using a flat reference function *r*(*δ*) = 1; **b** visualization of the FBST in Example 2 for the Bayesian two-sample *t* test for testing *H*_0_ : *δ* = 0 against the one-sided hypothesis *H*_1_ : *δ* < 0, using a wide half-Cauchy prior reference function *r*(*δ*) = *C*_+_(0,1)

The dimensions of *Θ* and ${{\varTheta }}_{H_0}$ are identical to Example 1, and the Bayesian *e* value $\overline {\text {ev}}(H_0) \approx 0.986$ expresses strong evidence against the null hypothesis *H*_0_ : *δ* = 0. Also, the standardized *e* value sev(*H*_0_) ≈ 0.001 < 0.05 is significant and leads to the same conclusion if a threshold of 0.05 is applied. The results are visualized in Fig. 4a. Figure 4b shows the FBST when a wide half-Cauchy prior *C*_+_(0,1) is used as the reference function *r*(*δ*) (Rouder et al., 2009)^7^. Figure 4a is produced by the code in Listing 12, where the additional parameter rightBoundary = 0 needs to be added to inform the plot() function that a one-sided hypothesis was used. Should the alternative be *H*_1_ : *δ* > 0, one would supply the argument leftBoundary = 0 to the plot() function. plot() function.

**Figure:**
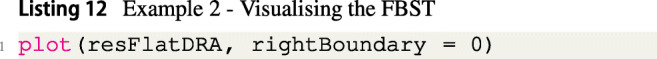

Based on the continuous quantification of evidence against *H*_0_ in form of $\overline {\text {ev}}(H_0)$ and the standardized *e* value sev(*H*_0_), one would reject the null hypothesis *H*_0_ : *δ* = 0 in favor of the alternative *H*_1_ : *δ* < 0. That is, the performance in the treatment group is better than in the control group which was not given directed reading activities.

## Example 3: Bayesian logistic regression

As a third example, it is demonstrated how to use the FBST via the fbst package in the context of the Bayesian logistic regression model (McElreath, 2020). Notice that while the focus is on the standard logistic model here, the procedure is applicable to any regression model of interest like probit or linear regression models. Data from the Western Collaborative Group Study (WCGS) of Rosenman et al., (1975) are used in which 3154 healthy young men aged 39 − 59 from the San Francisco area were assessed for their personality type. All were free from coronary heart disease at the start of the research. Eight and a half years later, the change in this situation was recorded. We use a subset of *n* = 3140 participants, where 14 participants have been excluded because of incomplete data. The data set is freely available in the faraway R package, so again first data is loaded and prepared as shown in Listing 13.

**Figure:**
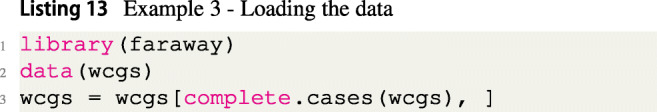

For illustration purposes, we use a Bayesian logistic regression model which studies the influence of the covariates age, height, weight, systolic blood pressure (sdp), diastolic blood pressure (dbp), fasting serum cholesterol (chol) and the number of cigarettes smoked per day (cigs) on the outcome chronic heart disease (yes/no, variable chd) stored in the response variable chd.

The model is fit via the Hamiltonian Monte Carlo sampler Stan (Carpenter et al., 2017; Kelter, 2020c), which uses the No-U-Turn sampler of Hoffman and Gelman (2014) to sample from the posterior distribution. The posterior distribution is obtained for the intercept and the seven regression coefficients *β*_1_,...,*β*_7_, belonging to the six covariates included in the model. The default weakly informative $\sigma \sim \exp (1)$ prior is assigned to the standard deviation *σ*, see Gabry and Goodrich (2020). The rstanarm package (Goodrich et al., 2020) is employed for fitting the Bayesian logistic regression model, and the code to prepare the data for Stan is given in Listing 14.

**Figure:**
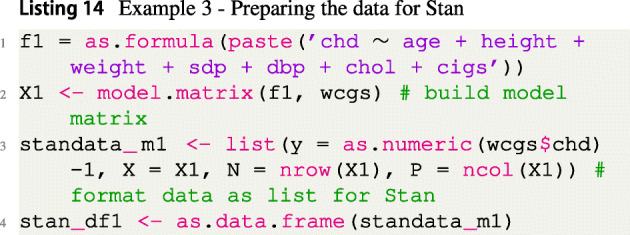

The standard weakly informative prior distribution $\beta _{j} \sim \mathcal {N}(0,2.5)$ is assigned to the regression coefficients *β*_*j*_,*j* = 1,...,7, and the intercept *β*_0_ is assigned the weakly informative default prior $\beta _{0} \sim \mathcal {N}(0,10)$ recommended by Gabry and Goodrich (2020). Listing 15 shows the code to fit the model via the rstanarm package, summarize, and plot the results.

**Figure:**
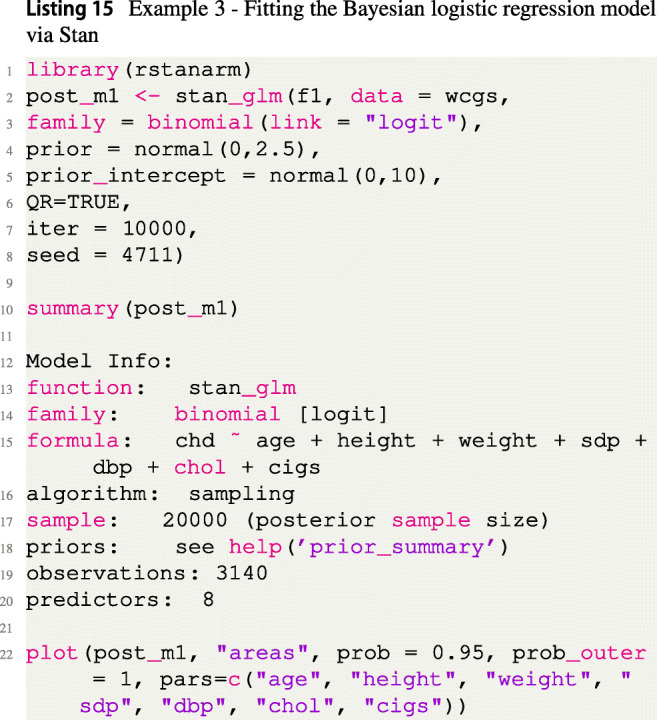

**Fig. 5:**
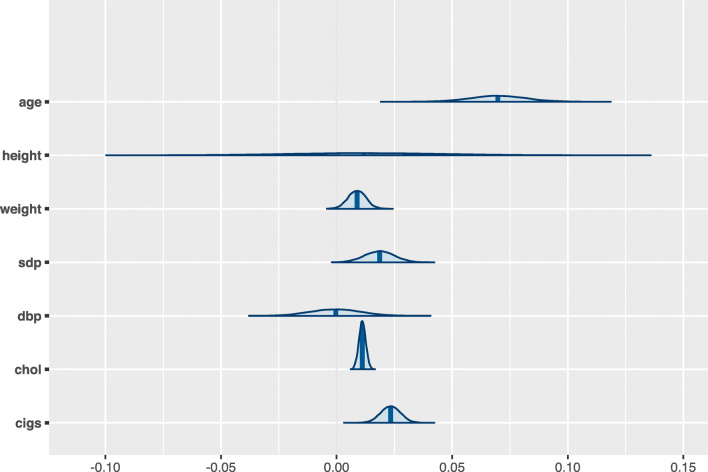
Marginal posterior distributions of the regression coefficients *β*_*j*_ in the Bayesian logistic regression model in Example 3

Figure 5 shows the marginal posterior distributions of the regression coefficients *β*_*j*_ for the Bayesian logistic regression model in Example 3.

To compute the FBST on the regression coefficients, one first needs to extract the posterior MCMC sample, as shown in Listing 16. For illustration purposes, the FBST is conducted on the regression coefficient belonging to the covariate weight. The FBST is computed using a normal prior $\mathcal {N}(0,2.5)$ as reference function, which was also used to fit the model. This way, the surprise function quantifies which parameter values *β*_*j*_ have been corroborated more by observing the data than the null value *β*_*j*_ = 0.

**Figure:**
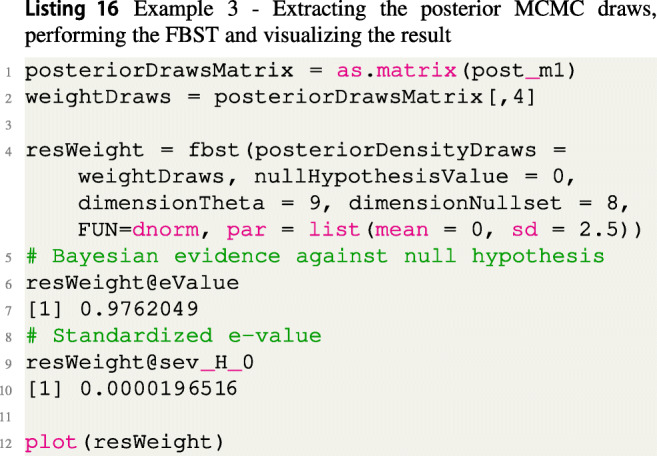

**Fig. 6:**
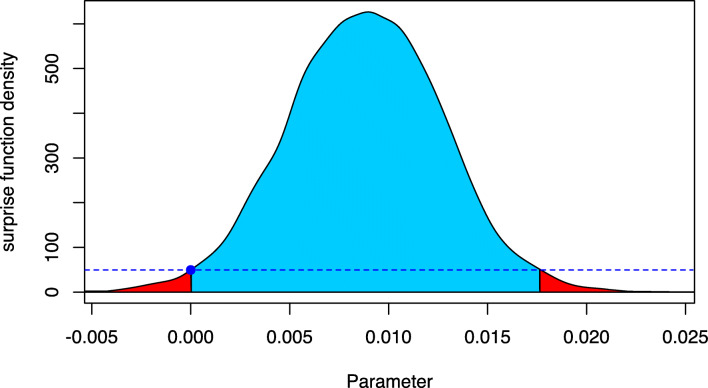
Visualization of the FBST for *H*_0_ : *β*_*j*_ = 0 against *H*_1_ : *β*_*j*_≠ 0 for the regression coefficient of the covariate weight in the Bayesian logistic regression model for the WCGS study

The results are also shown in Fig. 6, which is produced via the plot() function call in Listing 16.

Based on the standardized *e* value sev(*H*_0_) ≈ 0.0000196516 and the Bayesian evidence against *H*_0_, the *e* value $\overline {\text {ev}}(H_{0})\approx 0.9762$ one would reject the null hypothesis *H*_0_ : *β*_*j*_ = 0. Notice that because of seven predictors *β*_1_,...,*β*_7_, an intercept *β*_0_ and a standard deviation *σ* > 0 the parameter space *Θ* is nine-dimensional and the null set is eight-dimensional (one parameter coefficient *β*_*j*_ = 0 in ${{\varTheta }}_{H_{0}}$).

## Discussion

This paper introduced the R package fbst for computing the Full Bayesian Significance Test and the *e* value for testing a sharp hypothesis against the alternative. The conceptual approach and the statistical theory of the FBST were detailed, and three examples of statistical models frequently used in psychology and the biomedical sciences highlighted how the FBST can be computed in practice via the fbst R package. It was shown that both one-sided and two-sided hypotheses can be tested with the fbst package. The package’s core function fbst() requires only a posterior MCMC sample so it should be applicable to a wide range of statistical models used in the cognitive and biomedical sciences. The examples demonstrated that it is simple to combine the FBST via the fbst package with widely used libraries like rstanarm (Goodrich et al., 2020) or the BayesFactor package (Morey & Rouder, 2018). The provided summary and plot functions in the package allow intuitive use and produce appealing visualization of the FBST results which simplifies sharing and communication of the results with colleagues. Simulation studies were omitted in this paper because these were recently conducted by Kelter (2020a) to which the interested reader is referred. For more details on the theoretical properties of the FBST, see Pereira and Stern (2020).

To conclude, attention is directed to some limitations and possible extensions of the FBST and the fbst package presented in this paper. First, the fbst package is widely applicable but this strength can also be interpreted as a limitation. The fbst package requires a posterior distribution which has been derived analytically or numerically to conduct the FBST and compute the *e* value, so it is not a standalone solution.

Second, the core functionality in the current form is restricted to computing, summarizing and visualizing the FBST. Future extensions could include more detailed analysis results like robustness checks depending on the reference function used, see van Doorn et al., (2019). Also, in its current form the package uses only posterior MCMC draws, and future versions could provide the option to provide the posterior as a closed-form function. Another option to extend the functionality would be to make various algorithms available to estimate the posterior density based on the posterior draws: By now, only Gaussian kernel density estimation is used. In small sample situations the asymptotics of Bayesian posterior distributions guaranteed by the Bernstein-von-Mises theorem can be questionable and other approaches like spline-based interpolation or non-Gaussian kernels may be more useful.

Third, while the standardized *e* value may be used as a replacement of frequentist *p* values, it is also based on asymptotic arguments and future research is needed to study the behavior of the standardized *e* value sev(*H*_0_) for small samples. This is why in general it is recommended to prefer the continuous interpretation of the Bayesian *e* value $\overline {\text {ev}}(H_{0})$ over a threshold-oriented interpretation via the standardized *e* value sev(*H*_0_).

In closing, it must be emphasized that it is not argued against the appropriate use of *p* values, Bayes factors or any other suitable method of hypothesis testing. However, the ongoing debate about the concept of statistical significance shows that it is useful to explore existing alternatives for statistical hypothesis testing and investigate the relationships between these approaches both from a theoretical and practical perspective (Berger and Sellke, 1987; Makowski et al., 2019; Liao et al., 2020). The fbst R package introduced in this paper could contribute in particular to the former, as simulation studies can easily be carried out by employing the package, see for example Kelter (2020a).

There is much value in testing a sharp null hypothesis against its alternative in the cognitive sciences and psychology (Berger et al., 1994; Berger et al., 1997; Rouder et al., 2009; Kelter, 2020e). While there are also other useful approaches such as equivalence testing (Lakens, 2017; Lakens et al., 2018; Kruschke and Liddell, 2018b; Kruschke, 2018; Kelter, 2020d; 2020f) – the FBST has shown to be an attractive alternative to NHST and *p* values with desirable theoretical and practical properties (Kelter, 2020a; Pereira & Stern, 2020; Esteves et al., 2019). It is hoped that this package will be useful to researchers from the cognitive and biomedical sciences who are interested in a fully Bayesian alternative to null hypothesis significance testing which requires little methodological changes, but offers all the benefits of a fully Bayesian data analysis.

## Footnotes

1 Based on the analysis of Etz and Wagenmakers (2015) the Bayes factor goes back to Wrinch and Jeffreys (1921), and additionally, Haldane (1932) may have had more influence in the development of the Bayes factor than has hitherto been assumed.

2 If the posterior is available in closed form, one can directly sample from it and provide the argument with the samples.

3 However, a supplementary replication codebook is provided at the Open Science Foundation under https://osf.io/u6xnc/.

4 See for example Kelter (2020c) for a tutorial how to implement statistical models via Stan for the biomedical sciences. Another excellent resource is Kruschke (2015), who also shows how to obtain posterior MCMC draws for a variety of models in the cognitive sciences via the MCMC sampler JAGS (Plummer, 2003).

5 See www.jasp-stats.org

6 The data set is also provided as a .csv-file at the OSF repository https://osf.io/u6xnc/.

7 A *left*-half Cauchy prior is used, as under *H*_1_ : *δ* < 0, so a priori only negative effect sizes are assumed under the alternative hypothesis.
